# Insights into the Microbiological Safety of Wooden Cutting Boards Used for Meat Processing in Hong Kong’s Wet Markets: A Focus on Food-Contact Surfaces, Cross-Contamination and the Efficacy of Traditional Hygiene Practices

**DOI:** 10.3390/microorganisms8040579

**Published:** 2020-04-17

**Authors:** Patrick T. Sekoai, Shiqi Feng, Wenwen Zhou, Wing Y. Ngan, Yang Pu, Yuan Yao, Jie Pan, Olivier Habimana

**Affiliations:** 1 The School of Biological Sciences, The University of Hong Kong, Pokfulam, Hong Kong 999077, China; patricksekoai@gmail.com (P.T.S.); shiqi24@connect.hku.hk (S.F.); u3558681@connect.hku.hk (W.Z.); superguyhk@gmail.com (W.Y.N.); puyang@connect.hku.hk (Y.P.); yuan1222@connect.hku.hk (Y.Y.); 2 Institute for Advanced Study, Shenzhen University, Shenzhen 518060, China; panjie@szu.edu.cn

**Keywords:** Hong Kong’s wet markets, wooden cutting board, traditional hygiene practices, foodborne pathogens, biofilms

## Abstract

Hong Kong’s wet markets play a crucial role in the country’s supply of safe, fresh meat to satisfy the dietary needs of its population. Whilst food safety regulations have been introduced over the past few years to maintain the microbial safety of foods sold from these wet markets, it remains unclear whether the hygiene maintenance that is performed on the wooden cutting boards used for meat-processing is effective. In fact, hygiene maintenance may often be overlooked, and hygiene standards may be insufficient. If so, this may lead to the spread of harmful pathogens through cross-contamination, thereby causing severe risks to public health. The aim of this study was to determine the level of microbial transfer between wooden cutting boards and swine meat of various qualities, using 16S metagenomic sequencing, strain identification and biofilm screening of isolated strains. The results established that: (a) the traditional hygiene practices used for cleaning wooden cutting boards in Hong Kong’s wet markets expose the surfaces to potentially harmful microorganisms; (b) the processing of microbially contaminated meat on cutting boards cleaned using traditional practices leads to cross-contamination; and (c) several potentially pathogenic microorganisms found on the cutting boards have good biofilm-forming abilities. These results reinforce the need to review the traditional methods used to clean wooden cutting boards after the processing of raw meat in Hong Kong’ wet markets so as to prevent cross-contamination events. The establishment of proper hygiene protocols may reduce the spread of disease-causing microorganisms (including antibiotic-resistant microorganisms) in food-processing environments.

## 1. Introduction

Hong Kong’s wet markets play a central role in the country’s food sector by providing fresh foods to its population [1]. As opposed to meat from supermarkets, meat from wet markets is usually affordable and highly accessible [2]. However, food safety practices in these markets need to be thoroughly assessed as there has been a recent rapid increase in foodborne disease-related incidences in some Asian countries [3]. A recent study revealed that wet-market wooden cutting boards can pose a serious threat to consumers as they harbour various pathogens, including species that are associated with nosocomial infections such as *Klebsiella pneumoniae* [4]. The study also revealed the presence of antibiotic-resistant genes in the isolated bacterial strains [4]. 

The cleaning, maintenance and hygiene standards of wooden cutting boards used to process raw meat are often ignored by meat vendors in wet markets [5]. From a microbiological standpoint, the use of wooden cutting boards for processing raw meat may jeopardise public safety as wooden cutting board surfaces tend to attract various microbial contaminants. Consequently, the use of wooden materials in food processing has decreased over the past two decades as it has been shown that their use may increase the spread of foodborne pathogens. 

For example, a study by Dantas et al. [6] showed that the wooden cutting board surfaces were responsible for the transfer of microorganisms from poultry meat to cucumbers. The spread of *Salmonella enterica* serovar Enteritidis from poultry meat to cucumbers was assessed on different cutting board surfaces (wood, plastic, and glass) before and after washing of the surfaces. All of the tested strains were detected from unwashed cutting boards and cucumbers that had previously been cut and processed on such surfaces. The recovery of these strains on the cutting boards ranged between 10% to 100% following various washing routines, thus demonstrating the critical importance of correct hygiene practices for preventing the establishment of foodborne pathogens on food-preparation surfaces. 

Additionally, it has been observed that biofilm formation occurs more on wooden surfaces (60%) than on plastic (40%) or glass (10%) boards [6], thus reinforcing the importance of strict hygiene practices for wooden cutting boards. A similar study conducted in the cities of India also showed that most foods sold in these cities’ markets harboured pathogenic foodborne bacteria, such as Enterobacteriaceae, *Staphylococcus* spp. and *Bacillus cereus* [7]. Elsewhere, in the United States, other disease-causing microorganisms, such as *Escherichia coli*, were found on wooden cutting boards [8].

The wooden cutting board surface provides a suitable environment for the proliferation of diverse pathogens due to its porosity and hydrophilic properties [9,10]. Microbial contaminants thrive by taking advantage of the nutrients that are abundantly available during the processing of raw meat [11,12]. Therefore, wooden cutting boards may become a dangerous source of transferable disease-causing microorganisms if the necessary hygiene standards are ignored, as evidenced by these scientific reports. 

Food safety guidelines have been introduced in wet markets over the past few years, but food safety authorities focus primarily on the commercial processing of food products [13]. Hence, implementing proper hygiene procedures in wet markets may require the encouragement of knowledge exchange between those involved, as well as food safety training and detailed examination of vendors’ knowledge of safe raw meat-processing practice. It has been reported that in other African and Asian regions, many street food vendors do not have adequate knowledge with regards to food safety standards [14,15,16]. Therefore, failure to practically implement this knowledge will compromise the safety of consumers and the public as a whole. 

Unfortunately, microbial hazards associated with contaminated wooden cutting boards continue to be a problem in the wet markets of Hong Kong due to inadequate hygiene practices [4]. However, much is still unknown about the cross-contamination risks during meat processing using wooden cutting boards in Hong Kong’s wet markets. Therefore, it is crucial to identify the source of contamination, which may be the exposed meat products on display in the open air with no proper time- and temperature-controls, or the unhygienic surfaces of the wooden cutting boards, resulting from improper maintenance procedures, or a combination of both. 

The aims of this study were to determine the microbial contaminants on wooden cutting boards previously used for processing swine meat and to determine the efficiency of traditional cleaning routines used before processing meat. This was achieved by first identifying the microbiota of a previously used wet market cutting board before and after cleaning it using traditional methods, and determining the microbial composition of swine meat previously processed at wet markets and supermarkets, and that of imported swine meat. 

A variety of methods including traditional microbiological approaches and 16S and shotgun metagenomic sequencing analyses were used for assessing the microbial composition in terms of microbial community structure, biofilm-forming ability, and the presence of antibiotic-resistance genes. This study is expected to shed much needed light on the hygiene inadequacy of current wet-market wooden cutting board cleaning practices, thereby triggering dialogue between policymakers and stall keepers on matters related to improving food safety practices in Hong Kong’s wet markets. 

## 2. Materials and Methods 

### 2.1. Purchased Wooden Cutting Board and Swine Raw Samples

The wooden cutting board used in this project was purchased from Shek Tong Tsui Wet Market (Western District, Hong Kong) and had been previously used for the processing of raw meat. In such wet markets, wooden cutting boards are maintained using traditional cleaning procedures, which primarily involve the scraping of the entire surface of the wooden cutting board with a chopping knife until the top layer is removed, followed by rinsing the scraped surface with hot water. The word ‘traditional’ is used to reference the above mentioned cleaning procedures that are commonly used by Asian wet market vendors to clean these wooden cutting boards, as described in a previous study [4]. These cleaning techniques were believed to be efficient in removing contaminants until the introduction of proper hygiene practices by food safety authorities. Despite the implementation of adequate cleaning techniques, wet market vendors still rely on traditional cleaning methods. In the context of this study, the expression ‘meat processing’ refers to the action of chopping meat on a wooden cutting board as it is typically performed in Hong Kong’s wet markets. 

**Figure 1:**
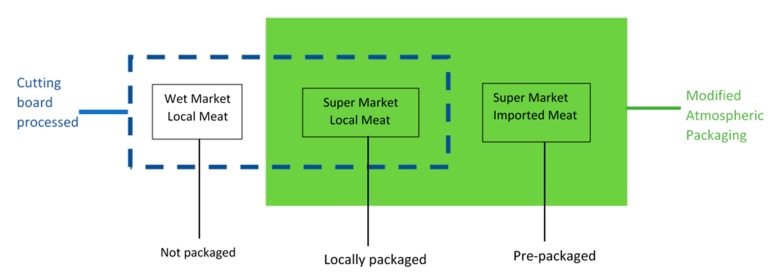
Schematic diagram illustrating the processing of meat samples on the cutting board.

Meat samples were acquired from areas surrounding the University of Hong Kong, Kennedy Town and Mong Kok. The studied pork samples were purchased from three markets, i.e., one sample was obtained from a nearby wet market stall and the other two samples were obtained from local supermarkets (YATA or ParkNShop). The meat samples (~200 g) purchased from the supermarkets were either processed and packaged at the supermarket or imported pre-packaged from Australia, Thailand or Canada (Figure 1). In this study, approximately 100 g of each meat sample was processed on the wooden cutting board. 

### 2.2. Sampling from the Wooden Cutting Board

Sampling from the wooden cutting board surface was performed as previously described by Lo et al. [4]. Briefly, pre-sterilised wooden-stick cotton swabs were hydrated with sterilized phosphate buffer solution and then used to swab a surface area equivalent to 10 cm^2^ on the most used part of the wooden cutting board. Swab sampling was performed on: (i) the selected area of the wooden cutting board prior to scraping; (ii) following scraping; and (iii) following scraping and rinsing with hot water. 

For bacterial enumeration and isolation, individual cotton swabs were homogenized in a test tube containing 9 mL of sterile Ringer’s solution, and serial dilutions (10^−6^ to 10^−16^) were made from the resulting homogenate. These dilutions were plated onto Tryptic Soy Agar (TSA) plates, and the plates were incubated at 30 °C for 20 h. Subsequently, colony counting and CFU estimation (Table 1) was performed by standard streaking on TSA plates (to support the growth of different species), and these were incubated at 30 °C for 20 h. Once colony homogeneity was established, by sub-culturing selected isolated colonies onto TSA plates, pure colonies were acquired by sub-culturing in sterile TSB medium (30 °C for 20 h). The resulting individual pure cultures were mixed with sterile 40% glycerol solution at a ratio of 1:1 and stored at −80 °C until further experiments. The remaining culture was used for isolate identification as described by Lo et al. [4] and as further described in Section 2.5.

For metagenomic profiling, separate swab samples were cultured aseptically in test tubes containing 5 mL of Bacto^TM^ TSB, and the resulting cultures were incubated at 30 °C for 20 h. A fraction of the resulting culture was used for DNA extraction and metagenomic profiling as described in Section 2.4.

### 2.3. Processing of Meat Samples on the Wooden Cutting Board

To determine the level of cross-contamination that could potentially occur because of the ineffective traditional cleaning methods, meat samples were processed and examined as described in Section 2.2. Briefly, the purchased meat samples were first divided into two equal parts; one piece (~100 g) was set aside as the control (i.e., unprocessed) and the other piece (~100 g) was processed on the wooden cutting board surface that had previously been subject to traditional cleaning method (Figure 1). The meat samples were processed on the wooden cutting board surface for 60 s and then systematically homogenized in stomacher bags to which 10 mL of sterile Ringer’s solution was added. Serial dilutions (10^−2^ to 10^−6^) of these mixtures were made and plated onto TSA plates, which were then incubated at 30 °C for 20 h. The resulting colonies were quantified for CFU estimation, and individual colonies were isolated by standard sub-streaking on TSA plates (30 °C for 20 h). Once colony homogeneity was established, by sub-culturing selected isolated colonies onto TSA plates, pure colonies were acquired by sub-culturing in sterile TSB medium (30 °C for 20 h). The resulting individual pure cultures were mixed with sterile 40% glycerol solution at a ratio of 1:1 and stored at −80 °C until further experiments. The remaining culture was used for isolate identification as described by Lo et al. [4] and as further described in Section 2.5.

For metagenomic profiling, a 100 µL homogenized suspension was first inoculated in 9.9 mL TSB medium before incubation at 30 °C for 20 h. A fraction of the resulting culture was used for DNA extraction and metagenomic analysis as outlined in Section 2.4. The control experiments (unprocessed meat) were also performed as described in this section. 

### 2.4. DNA Extraction and 16S Metagenomic Sequencing

Genomic DNA was extracted from overnight TSB cultures using the PureLink™ Microbiome DNA Purification Kit (#A29790; Invitrogen™, Carlsbad, CA, USA), according to the manufacturer’s instructions. Prior to sequencing, the concentration and purity of genomic DNA were verified using a BioDrop Duo spectrophotometer (BioDrop, Cambridge, UK). For 16S metagenomic analysis, DNA samples were sent to Novogene (Shenzhen, China) for PCR-free library construction and sequencing (16S V3-V4 region) on a HiSeq-PE250 platform. The resulting 16S rRNA gene libraries were amplified using the published primer set: 27F (AGAGTTTGATCMTGGCTCAG) and 1492R (GGTTACCTTGTTACGAC TT) [17]. Metagenomic analysis was performed on cleaned raw sequence reads following quality control and filtering of barcodes and primer sequences. The effective data was used to determine the operational taxonomic units (OTU) cluster and species annotation required for establishing relative species, evenness and abundance distributions. 

The obtained paired-end reads were merged using fast length adjustment of short reads (FLASH) [18] following the removal of barcodes and primer sequences. Quality filtering was performed on raw reads using specific filtering conditions to obtain high-quality clean reads [19] according to the QIIME quality control process [20]. The obtained effective tags were compared with a reference database (Gold database) using the UCHIME algorithm [21] to remove chimeric sequences. Sequence analysis was performed using the UPARSE algorithm [22], wherein sequences with a similarity index of ≥ 97% were assigned to the same OTUs. To obtain a representative sequence for each OTU, screening for species annotation was performed using the GreenGene Database [23] based on the RDP Classifier [24]. The phylogenetic relationships of different OTUs, the differences between dominant species in samples (groups), and all multiple sequence alignments were analysed using PyNAST v1.2 [20] against the “Core Set” dataset in the GreenGene database.

### 2.5. Isolate Identification Via 16S rRNA Sequencing

Isolated strains previously stored at −80 °C were first reactivated in TSB and then incubated at 30 °C for 20 h. Subsequently, the genomic DNA of strains was extracted from monocultures as described in Section 2.4. Twenty-one genomic DNA isolates were obtained from the wooden cutting board and 35 isolates from the meat samples. These were sent to the Centre for Genomic Sciences (Li Ka Shing Faculty, HKU, Hong Kong) for PCR amplification of the 16S rRNA gene, followed by Sanger DNA sequencing using an Applied Biosystems 3730xl DNA Analyzer [25]. The forward and reverse primers used are described in Section 2.4. A nucleotide BLAST DNA search was then performed in the DNA database software of the NIH (United States) using the amplicon sequences identified for the isolates from the website: www.ncbi.nlm.gov. 

### 2.6. Biofilm Screening of Identified Bacterial Isolates

**Figure 2:**
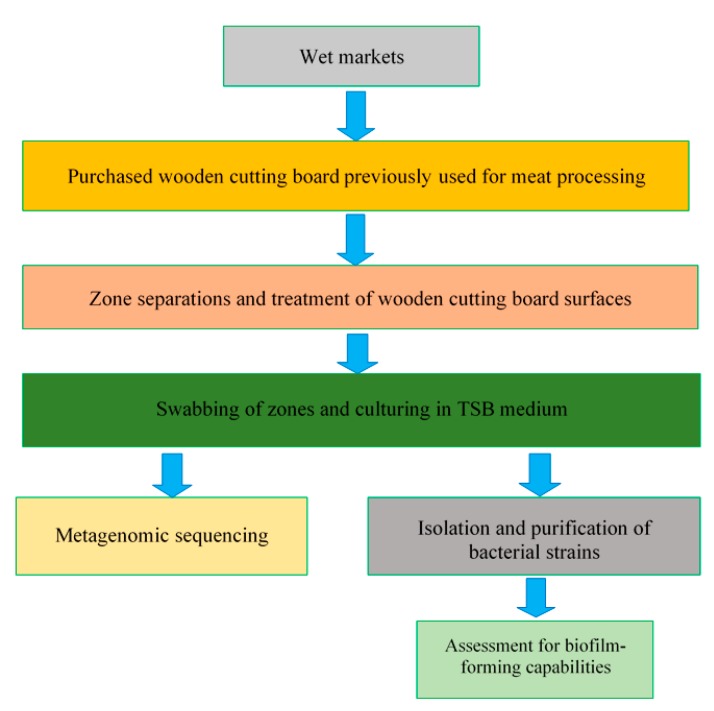
Schematic representation of the experimental plan.

Following the identification of isolated strains, individual strains were reactivated, sub-cultured in TSB and incubated at 30 °C for 20 h. To compare the biofilm-forming abilities of the selected individual strains, bacterial suspensions were first standardised by optical density measurements, and then 100 µL of each bacterial suspension was inoculated in TSB in a ratio of 1:100 in the individual wells of a 96-well plate, with four replicate wells created for each species. The screening of biofilms was conducted as outlined by O’Toole [26]. Briefly, after incubation, the suspended cells in each well were removed, and the wells were rinsed with distilled water. Thereafter, 125 µL of 0.1% crystal violet solution was added to the emptied wells to bind to biofilm-forming species present on the well walls. The crystal violet solution was then removed from the wells after 10 min, and a second rinse with distilled water was performed. Residual crystal violet was solubilized with 125 µL of 30% acetic acid solution, and the absorbance of solution in each well was measured at 550 nm. Control wells containing sterile TSB were treated by the same staining and rinsing procedure. The experiments were conducted in duplicate. Moreover, all of the experimental procedures conducted in this study (Section 2.1, Section 2.2, Section 2.3, Section 2.4, Section 2.5 and Section 2.6) are summarised in Figure 2. 

## 3. Results

### 3.1. Quantification of MicroorGanisms Found on the Wooden Cutting Board and in Meat Samples 

The microbial loads of the wooden cutting board (which had previously been used for meat processing in Hong Kong’s wet markets) and processed meat samples were quantified by estimating the colony-forming units per surface area for the cutting board surfaces (CFU/cm^2^) and the colony-forming units per gram (CFU/g) for the meat samples, as shown in Table 1. The cutting board was found to harbour a microbial load of 6.35 log_10_ CFU/cm^2^, despite cleaning it with traditional hygiene procedures. In addition, a generalized microbial load increase was observed in processed meat samples from wet supermarkets and imported meats by 4.26 log_10_ CFU/g and 4.29 log_10_ CFU/g, respectively. However, these results serve as a preliminary microbial quantification study in the context of this work. Therefore, in-depth microbial profiling studies were conducted in Section 3.2 and Section 3.3 to identify the types of microbial contaminants found on the cutting board.

### 3.2. Microbial Community Profile on the Wooden Cutting Board Surfaces 

**Figure 3:**
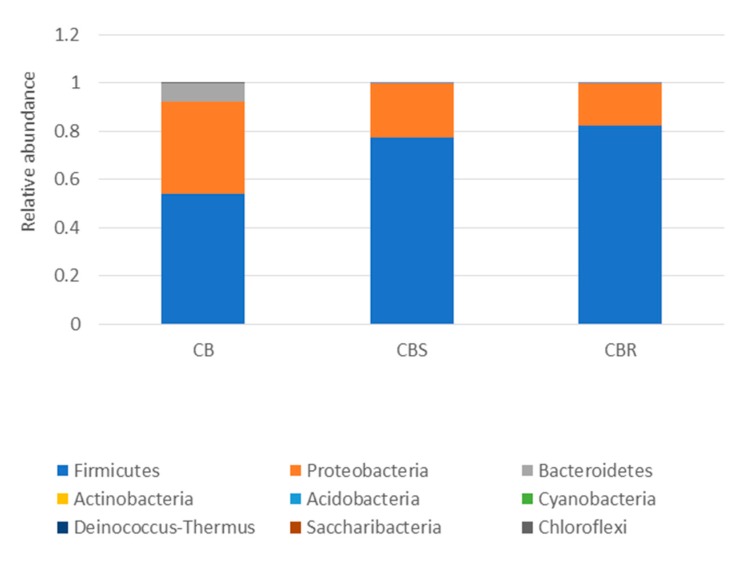
Taxonomic community assignments (at the phylum level) of the swab samples collected from the purchased cutting board (CB), the scraped cutting board (CBS) and the rinsed cutting board (CBR), respectively.

**Figure 4:**
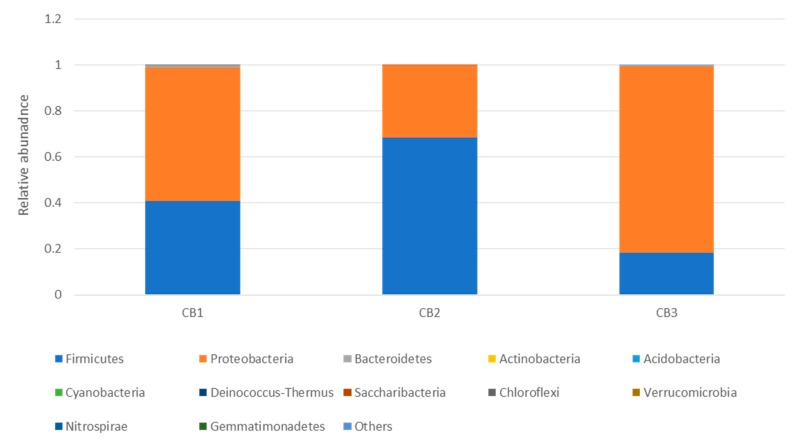
Taxonomic community profiles (at the phylum level) of the swab samples obtained after performing the first (CB1), second (CB2) and third (CB3) cleaning routines on the cutting board.

The 16S metagenomic sequencing data showed that the microbial population of the wooden cutting board consisted mainly of microorganisms from the phyla Firmicutes and Proteobacteria (Figure 3). Further taxonomic analysis at the species-level revealed the prevalence of *Lactococcus garvieae*, *Weissella hellenica* and *Kurthia gibsonii* on the wooden cutting board (Supplementary Figure S1). The efficacy of the traditional hygiene practices, which involve the scraping and rinsing of the wooden cutting boards, was also investigated by examining the microbial profile after the wooden cutting board was subjected to three consecutive cleaning routines, as shown in Figure 4. Notably, *W. hellenica* and *L. garvieae* remained the most prevalent bacterial contaminants on the cutting board after these cleaning routines (Supplementary Figure S2). 

**Figure 5:**
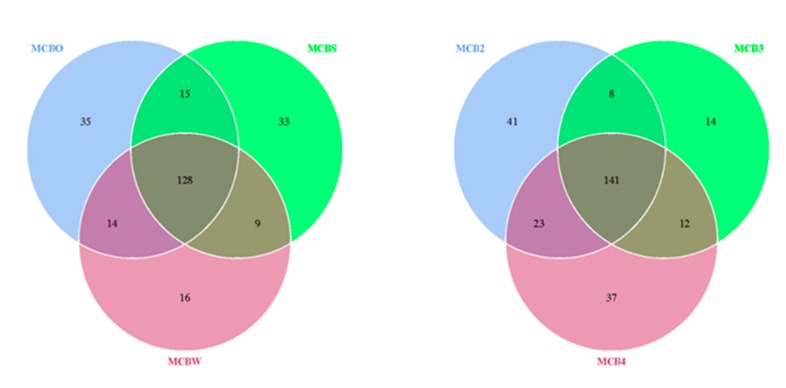
Venn diagram showing the degree of overlap of bacterial operational taxonomic units (OTUs) between the swab samples obtained from the purchased cutting board (MCBO), the scraped cutting board (MCBS) and the rinsed cutting board (MCBW). MCB2, MCB3 and MCB4 represent sampling after the second, third and fourth experimental repetitions, respectively. The numbers of shared and unique OTUs between bacterial species are shown.

A beta-diversity analysis was performed to evaluate the extent of the unique and shared OTUs between the bacterial species detected on the untreated and cleaned wooden cutting board (cleaned using the traditional methods), as shown in Figure 5. It was revealed that the traditional hygiene methods used to clean the wooden cutting board did not affect the microbiological load, with 128 OTUs shared between the treated and untreated cutting boards. Surprisingly, the repeated cleaning procedures did not affect the outcome of the microbiological load. Here, 141 OTUs were shared between the samples following the traditional cleaning routines. In addition to these results, an antibiotic-resistance gene profile analysis was conducted by screening the genomic DNA against a ResFinder database, as described by Lo et al. [4]. The analysis revealed a high number of positive hits for resistance genes against various classes of antibiotics (Supplementary Table S1). Genes associated with resistance to aminoglycosides, beta-lactams, fluoroquinolones, MLS-macrolides, lincosamide, streptogramin B, phenicol, tetracycline, and trimethoprim were detected in all treated wooden cutting board samples.

### 3.3. Microbial Community Profile in Meat Samples 

**Figure 6:**
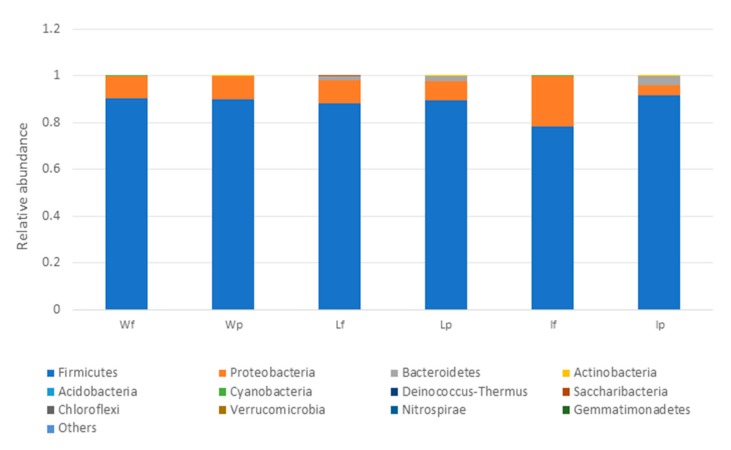
Relative abundance profiles of meat samples (at the phylum level) with Lf, If and Wf representing the unprocessed local meat, unprocessed imported meat and unprocessed wet market meat, respectively. LP, Ip and Wp represent the processed local meat, processed imported meat and processed wet market meat, respectively.

The microbial profiles of the processed meat (local meat (Lp), imported meat (Ip), and wet market meat (Wp)) and unprocessed meat (unprocessed local meat (Lf), unprocessed imported meat (If), and unprocessed wet market meat (Wf)) were obtained by analysing the 16S metagenomic amplicon sequencing data. Firmicutes was the most dominant phylum in all of the meat samples, but an increase in the relative abundance of this bacterial group was also observed after the processing of imported meat (Ip) on the wooden cutting board (Figure 6). At the species level, *L. garvieae* and *W. hellenica* were the predominant bacterial species in most samples, including the unprocessed meat (Wf and Lf) (Supplementary Figure S3). 

### 3.4. Biofilm Assessment of Identified Bacterial Isolates

**Figure 7:**
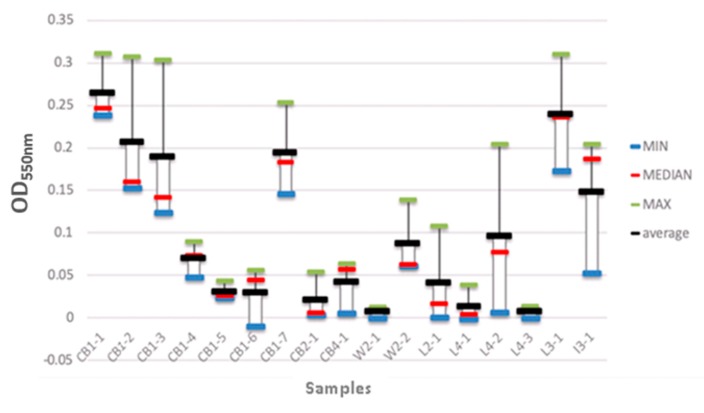
Biofilm biomass of the isolated bacterial strains. CB1-1 to CB1-7 represent the bacterial strains that were isolated from the wooden cutting board after the first week of incubation. CB2-1 represents the strains that were isolated from the cutting board after the second week of incubation. CB4-1 represents the strains that were isolated from the cutting board after the fourth week of incubation. W2-1 and W2-2 represent the strains that were isolated from the wet market meat samples after the first and second week of incubation, respectively. L2-1 and L3-1 represent the bacterial strains that were isolated from the local meat after the second and third week of incubation, respectively. L4-1 to L4-3 represent the bacterial strains that were isolated from local meat after the fourth week of incubation. I3-1 represents the bacterial strains that were isolated from imported meat after the third week of incubation.

The ability of the isolated bacterial strains to form biofilms was also evaluated (Figure 7). Seventeen isolates were grown in 96-well plates, and the resulting biofilms were analysed spectrophotometrically. The strains that were isolated from the wooden cutting board surfaces (CB1-1 to CB1-3) showed a higher biofilm-forming capability, i.e., the average biofilm biomass values ranged from 0.18 to 0.28. The strains isolated from the local meats (L3-1 and L4-2) also exhibited high biofilm-forming ability, with biofilm biomass values of 0.24 and 0.10, respectively. In contrast, the bacterial strains isolated from the wet market meats (W2-1 and W2-2) had the lowest biofilm-forming capability, with biofilm biomass values of 0.0 and 0.08, respectively. 

The biofilm-forming isolates were screened (as outlined in Section 2.5), and were identified as: *Pantoea* sp. (CB1-1), *Borreliella chilensis* (CB1-2), *Escherichia coli* O113:H21 FWSEC0011 (CB1-3), *Escherichia coli* E26-48 (CB1-4), *Escherichia coli* 602354 (CB1-5), *Pseudomonas lundensis* (CB1-6), *Proteus mirabilis* (CB1-7), *Aeromonas dhakensis* (CB2-1), *Aeromonas salmonicide* (CB4-1), *Myroides phaeus* sp. (W2-1), *Serratia marcescens* BWH-35 (W2-2), *Citrobacter* sp. LY-1 (L2-1), *Klebsiella pneumoniae* (L3-1), *Salmonella enterica* CFSAN064033 (L4-1), *Escherichia coli* (L4-2), *Citrobacter freundii* (L4-3), *Pseudomonas aeruginosa* (I3-1). 

## 4. Discussion

### 4.1. The Influence of Traditional Cleaning Practices on the Wooden Cutting Board

The results showed that the traditional cleaning procedures were ineffective as representative species of the phyla Firmicutes and Proteobacteria were still detectable on the wooden cutting board surfaces after these cleaning procedures (Figure 3 and Figure 4). Moreover, *W. hellenica* and *L. garvieae* remained the most prevalent bacterial contaminants on the cutting board after these cleaning routines (Supplementary Figure S2). One study reported that microbial contaminants on a wooden cutting board can be removed by vigorous washing and rinsing routines [8]. Nevertheless, some microbial species could still be present on the cutting board after such treatment because diverse microbial communities colonize the porous surfaces of wooden cutting boards and usually form resilient and hazardous biofilms. The abundance of these phyla may be attributable to the ubiquity of microorganisms belonging to these groups and to the resistance of some bacterial species to antibacterial treatments, particularly endospore-forming bacteria of the phylum Firmicutes [27,28]. The increased prevalence of Firmicutes may also increase the likelihood of cutting boards being exposed to pathogenic species of this phylum. 

The presence of *L. garvieae*, an important zoonotic pathogen in humans and aquaculture [29], on the cutting board suggests that cross-contamination occurred due to improper hygiene routines used for the cutting boards or utensils or by the vendor in seafood processing. However, further investigation is warranted to establish the link between the cutting board flora, human contact and the proximity of seafood stalls at wet markets. 

Notably, *W. hellenica* is a lactic acid-producing bacterium that is found in diverse habitats such as foods (meat, vegetables and fruits), humans and animals [25]. The nutrients that were trapped on the porous surfaces of the wooden cutting board might have been utilised by *W. hellenica* for its growth requirements given its ubiquity and the ability to breakdown complex polysaccharides [30]. In addition, this microorganism belongs to the phylum Firmicutes [31,32]. 

*K. gibsonii* also belongs to the phylum Firmicutes [33,34]. However, its presence on the cutting board was surprising because it is usually associated with sexually transmitted zoonotic infections [35,36]. This finding also suggest that cross-contamination had occurred via human vectors attributable to the high daily traffic of customers buying meat products and simultaneously spreading various pathogenic species. Furthermore, the proximity of wet markets to nearby clinics and hospitals might have also contributed to the transfer of these microorganisms, as shown in other related studies [4,35,36]. The above mentioned bacterial species were also identified in other food processing studies and were reported to be the primary cause of various foodborne illnesses [37,38,39]. Further epidemiological surveys would help validate the presence of such pathogens on wet market wooden cutting boards. The detection of pathogenic species on the wooden cutting board surfaces even after it was subjected to traditional cleaning practices is worrisome, as it indicates that such cutting boards may contribute to the spread of disease-causing illnesses in Hong Kong, as mentioned earlier. Therefore, these ineffective hygienic methods need to be critically reviewed, and proper hygienic practices must be urgently implemented to guarantee the food safety of meat sold in the wet markets of Hong Kong. 

The presence of such a variety of Antibiotic Resistant Genes (ARGs) on the cutting board used for food processing should be a matter of great concern for health professionals and policymakers. Although the origin of these acquired ARGs could not be elucidated based on our observations, these results suggest that the re-evaluation of wooden cutting board cleaning practices in wet markets should be a public health priority. The meat products purchased from these wet markets undergo additional thermal processing at other sites, and thus, no information is available regarding the spread of these ARGs and clinically relevant strains from wet markets and residences due to poor kitchen hygiene practices. This matter is especially concerning for people at a higher risk of incurring adverse outcomes from exposure to microorganisms harbouring ARGs. 

Therefore, future studies should investigate the transfer of cutting board microorganisms and associated ARGs onto freshly cut meat products. Moreover, wet markets should be extensively surveyed to determine the risk of cross-contamination. Further investigations should also examine the microbial ecology of wooden cutting boards used in these wet markets to determine the prevalence of clinically relevant microorganisms such as *Enterococcus faecalis* in the multi-species biofilm consortia, as well as the transfer and spread of ARGs. 

### 4.2. Microbial Community Profile in Meat Samples 

The prevalence of Firmicutes in unprocessed meats may be an indicator of the samples’ previous handling and the level of hygiene at the time and point of purchase. Typically, supermarkets also process their meat products using wooden cutting boards prior to packaging and storage, usually under controlled temperature and relative humidity conditions. In sharp contrast, the apparently unhygienic surface conditions of the wooden cutting boards in wet markets, in combination with Hong Kong’s high ambient temperature and high relative humidity conditions, are conducive to pathogenic microbial proliferation on freely displayed meat products [4]. It has been reported that certain bacteria belonging to phylum Firmicutes can survive at low temperature [40,41]. For example, some species belonging to this bacterial group have survival mechanisms to withstand harsh environmental conditions [42], such as by producing endospores at low temperatures, which then remain dormant until conditions become favourable [43,44]. Therefore, it is likely that these microorganisms were also present in the refrigerated meat samples before being processed on the wooden cutting board surface. Because of the aciduricity and ability of these microorganisms to use a wide range of nutrients, the detection of *L. garvieae* and *W. hellenica* was unsurprising, and these findings adds to the growing scientific evidence of the ubiquity of these species [45,46,47]. Similarly, the prevalence of *Enterococcus faecalis* in the unprocessed imported meat also highlights the complex pathogenic profile of the species inhabiting these meat products. *E. faecalis* is usually associated with nosocomial infections and has been reported as being one of the most challenging pathogens to treat due to its drug resistance [48,49].

Given that the presence of ARGs on the wooden cutting board has been established, future studies should be conducted to determine the microorganisms responsible for harbouring and spreading such ARGs. Other microbial contaminants, such as *Weissella ceti*, *Macrococcus caseolyticus*, and *Lactococcus raffinolactis*, were also detected in these meat samples, although in smaller fractions (Supplementary Figure S3). In addition to the problem of cross-contamination, other factors might have contributed to the prevalence of pathogens in these wet markets, such as the lack of temperature control, fluctuations in relative air humidity, and open-air exposure of meat products during display and storage [4], as highlighted earlier.

### 4.3. The Biofilm-Forming Capability of the Isolated Bacterial Strains

These results showed that the good porosity and hydrophilicity of the wooden cutting board surfaces provided favourable conditions for the growth of biofilm-forming communities. The nutrients (meat pieces) that remain attached to the cutting board surfaces after the processing of raw meat may also enhance the development of multi-species biofilms. Notably, such resident microorganisms have been reported to synergistically interact with one another, which strengthens their modes of survival [50]. For example, strains from poor biofilm-forming species have been found to combine with strains from strong biofilm-forming species to form complex biofilm communities [51,52]. Furthermore, the atmospheric conditions (warm ambient temperatures and high humidity) and the large number of people who regularly buy meat from these wet markets favour the adhesion of these resilient, pathogenic microorganisms on the cutting board surfaces and their biofilm-formation mechanisms [53,54], as elucidated earlier. 

Similar conclusions were drawn by Lim et al. [55] when assessing biofilm formation in a cafeteria kitchen. They found that most of the biofilm communities belonged to the phylum Firmicutes, with *Bacillus*, *Staphylococcus*, *Acinetobacter* and *Kocuria* being the dominant genera. These authors also observed that some of these bacterial species were resistant to certain types of disinfectants [55], which implies that robust, well-validated hygiene regimes should be used for cleaning food processing environments. Weber et al. [56] studied the bacterial composition of biofilms in bovine milk and found that Firmicutes, Proteobacteria, Actinobacteria, and Bacteroidetes were the dominant phyla. In the phylum Actinobacteria, *Rhodococcus*, *Dermacoccus*, *Gordonia*, *Kocuria* and *Microbacterium* were the main genera. In the Firmicutes phylum, *Bacillus*, *Lactococcus* and *Staphylococcus* were the most prevalent genera. *Acinetobacter* was the most common genera in the phylum Proteobacteria, and *Chryseobacterium* was the most detected genera in the phylum Bacteroidetes. In another study, Sharma and Anand [57] examined the biofilms in milk processing pipelines and observed that the bacterial contaminants consisted mainly of *Bacillus* before and after the pasteurisation stages. 

In summary, the hazardous effects of biofilms on wooden cutting board surfaces should not be overlooked because improper hygiene procedures in food processing environments could lead to the spread of foodborne illnesses in humans and economic losses. Furthermore, understanding the ecology of microbial biofilms in food processing environments is essential to the establishment of effective sanitisation methods. Moreover, it should be noted that the presented findings in this study using TSB or TSA media only reflects a fraction of what could be identified and isolated from the wooden cutting board and meat samples. Therefore, future research should consider the use of other culture media to study the full scope of transferable bacteria between the wooden cutting boards and meat samples.

## 5. Conclusions

The present study aimed to provide deeper insights into the microbial communities associated with the wooden cutting boards used for the processing of raw meat, in Hong Kong’s wet market. This was achieved by using a variety of methods including traditional microbiological techniques and 16S and shotgun metagenomic sequencing methods. The results obtained from this study were alarming because diverse pathogenic species, including strains that are associated with clinical infections, were isolated from the wooden cutting board surfaces. It was also observed that the traditional hygiene methods were not effective in the eradication of microbial contaminants as hazardous biofilm-forming species were found on the cutting board surfaces. In addition, the porosity and hydrophilicity of the wooden cutting board surfaces, coupled with the warm and humid atmospheric conditions of Hong Kong, were also shown to favour the spread of disease-causing illnesses in food-processing environments in Hong Kong. 

Based on these results, the need to evaluate the hygiene methods used in the wet markets of Hong Kong cannot be over-emphasised, especially given that SARS-CoV-2 may have originated in a wet market in Wuhan, China [58,59]. To prevent the spread of foodborne pathogens in these markets, several strategies are proposed in this study. First, the wet market vendors must be educated about proper hygiene methods that they should use to reduce the spread of pathogens in their food-processing environments. Second, food safety authorities should regularly monitor these wet markets to guarantee the safety of meat sold to consumers. Lastly, more studies should be conducted to understand the sources of some of the pathogenic species isolated in this study, especially those related to nosocomial infections.

## Figures and Tables

**Table 1:** Colony counts for the wooden cutting board and meat samples.

Sample	Colony Count
Fresh meat from the wet market	1.90 × 10^4^ ± 2.25 × 10^4 a^
Processed meat from the wet market	1.85 × 10^4^ ± 6.17 × 10^3 a^
Fresh meat from the local supermarket	9.31 × 10^2^ ± 7.44 × 10^2 a^
Processed meat from the local supermarket	9.02 × 10^3^ ± 8.42 × 10^3 a^
Imported fresh meat	8.1 × 10^1^ ± 6.3 × 10^1 a^
Imported processed meat	1.98 × 10^4^ ± 2.0 × 10^3 a^
Purchased wooden cutting board	2.26 × 10^6^ ± 5.37 × 10^5 b^

^a^ CFU/g, ^b^ CFU/cm^2^.

## Supplementary Materials

The following are available online at https://www.mdpi.com/2076-2607/8/4/579/s1, Figure S1: Taxonomic community profiles (at the genus level) of the swab samples collected from the purchased cutting board (CB), the scraped cutting board (CBS), and the rinsed cutting board (CBR), respectively, Figure S2: Taxonomic community profiles (at a genus level) of the swab samples following the second (CB2), third (CB3), and fourth (CB4) cleaning routine on the cutting board, Figure S3: Relative abundance profiles of meat samples (at a genus level) with Lf, If, and Wf representing the unprocessed local meat, unprocessed imported meat, and unprocessed wet market meat, respectively. LP, Ip, and Wp represents the processed local meat, processed imported meat, and processed wet market meat, respectively, Table S1: ResFinder resistome summary results following cutting board treatments.
