# Supplementary material for: Insights into the Microbiological Safety of Wooden Cutting Boards Used for Meat Processing in Hong Kong’s Wet Markets: A Focus on Food-Contact Surfaces, Cross-Contamination and the Efficacy of Traditional Hygiene Practices
Source: Microorganisms. 2020 Apr 17;8(4):579. doi: 10.3390/microorganisms8040579 (PMC7232214; doi:10.3390/microorganisms8040579)
Supplement: Supplementary file 1 [file microorganisms-08-00579-s001.pdf]

Article

Insights into the Microbiological Safety of Wooden Cutting Boards Used for Meat Processing in Hong Kong's Wet Markets: A Focus on Food-Contact Surfaces, Cross-Contamination and the Efficacy of Traditional Hygiene Practices

Patrick T. Sekoai [1], Shiqi Feng [1], Wenwen Zhou [1], Wing Y. Ngan [1], Yang Pu [1], Yuan Yao [1], Jie Pan [2] and Olivier Habimana [1,]*

[1] The School of Biological Sciences, The University of Hong Kong, Pokfulam, Hong Kong 999077, China; patricksekoai@gmail.com (P.T.S.); shiqi24@connect.hku.hk (S.F.); u3558681@connect.hku.hk (W.Z.); superguyhk@gmail.com (W.Y.N.); puyang@connect.hku.hk (Y.P.); yuan1222@connect.hku.hk (Y.Y.)

[2] Institute for Advanced Study, Shenzhen University, Shenzhen 518060, China; panjie@szu.edu.cn

* Correspondence: ohabim@hku.hk

Received: 17 March 2020; Accepted: 15 April 2020; Published: 17 April 2020

Supplementary Materials:

	Resistance genes	Samples		
		Control	WM	CIP
Aminoglycoside	aac(3)-IId	X	X	X
	aac(6')-aph(2'')	X	X	X
	aadA1			X
	aadA2			X
	aac(6')-Ib-cr	X	X	X
	aph(3')-Ia	X	X	X
	aph(3')-IIa	X		X
	aph(3'')-Ib		X	X
	aac(6')-Ic		X	X
	aadA5		X	X
	str	X	X	X
	aac(6')-Iaa			X
	aadA16	X	X	X
	aph(6)-Id	X	X	X
	aph(4)-Ia	X	X	
	ant(6)-Ia	X		
	aadA24	X	X	
	aadA2b	X	X	
	aac(3)-IV	X	X	
Beta-lactam	blaTEM-1B	X		X
	blaACT-7	X	X	X
	blaCTX-M-69			X

	Resistance genes	Samples		
		Control	WM	CIP
MLS - Macrolide, Lincosamide and Streptogramin B	mdf(A)	X	X	X
	mph(A)	X		X
	lnu(A)	X	X	X
	lnu(F)	X	X	
	lsa(A)	X		
	mef(B)	X	X	
	mph(A)	X	X	
	erm(B)	X	X	
	mdt(A)		X	
Phenicol	catA1	X	X	X
	cmlA1	X	X	X
	floR	X	X	X
	catA2	X		
	catB3		X	
Fluoroquinolone	aac(6')-Ib-cr	X	X	X
	qnrD1	X	X	X
	qnrB6	X		X
	oqxA	X	X	X
	qnrS1	X	X	X
	oqxB	X	X	X
	qnrB2	X		
	qnrS2	X	X	

blaCMY-89			X
blaSHV-185	X		X
blaCMY-101			X
blaCTX-M-14			
blaOXA-10	X	X	X
blaCMY-65	X		
blaCMY-82	X		
blaCTX-M-3	X		
blaOXA-1			
blaCTX-M-196		X	
blaSHV-33		X	
blaCMY-105		X	
blaCTX-M-55		X	
blaCMY-70		X	
qnrB5	X		
qnrB52		X	

Table S1: ResFinder resistome summary results following cutting board treatments**:** The surface layer of the three treated areas was scraped, and the scraped wooden material was inoculated into sterile TSB, as previously described by Lo et al (2019) (Lo et al., 2019). Three samples were identified control (no treatment), wet market treatment (scrapping), and CIP (0.1% sodium hypochlorite). The scrapped layers were used as inoculants in TSB. Following incubation at 25°C for 20 hours, genomic DNA extraction was performed on overnight cultures as described in section 2.2.1. The genomic DNA samples were then sent for shotgun metagenomic sequencing at BGI (Shenzhen) and sequenced on BGISEQ-500 (BGI, Shenzhen). ResFinder 3.1 was employed for the identification of acquired antimicrobial resistance genes ARG in analysed samples (Zankari et al., 2012). Before using ResFinder 3.1, the metagenomics data was pre-treated in the following steps: dereplication, trimming (threshold Q value: 25), fasta file extraction, pairing of short reads, and finally SPades assembly. The assembled contigs were then uploaded to ResFinder 3.1. All parameters followed the default setting of ResFinder 3.1. Briefly, identity threshold between the best matching resistance gene in the database and the corresponding sequence in the genome was 90%. The minimum percentage of a sequence that overlaps the resistance gene to count as a hit for that gene was set at 60%.

1. Lo, M. Y., Ngan, W. Y., Tsun, S. M., Hsing, H. L., Lau, K. T., Hung, H. P., Habimana, O. (2019). A Field Study Into Hong Kong's Wet Markets: Raised Questions Into the Hygienic Maintenance of Meat Contact Surfaces and the Dissemination of Microorganisms Associated With Nosocomial Infections. *Front Microbiol, 10*, 2618. doi:10.3389/fmicb.2019.02618
2. Zankari, E., Hasman, H., Cosentino, S., Vestergaard, M., Rasmussen, S., Lund, O., . . . Larsen, M. V. (2012). Identification of acquired antimicrobial resistance genes. *Journal of Antimicrobial Chemotherapy, 67*(11), 2640-2644.

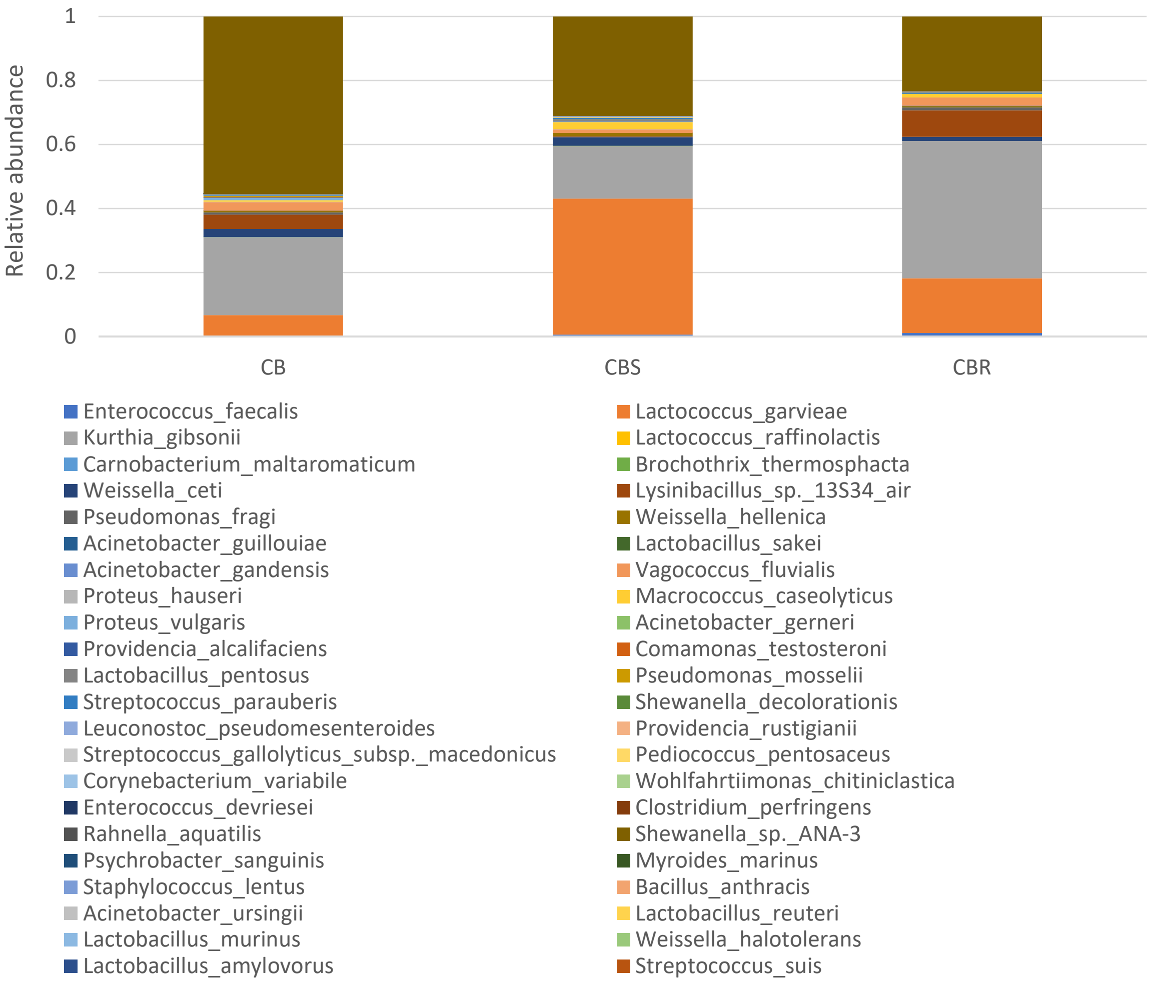

Figure S1: Taxonomic community profiles (at the genus level) of the swab samples collected from the purchased cutting board (CB), the scraped cutting board (CBS), and the rinsed cutting board (CBR), respectively.

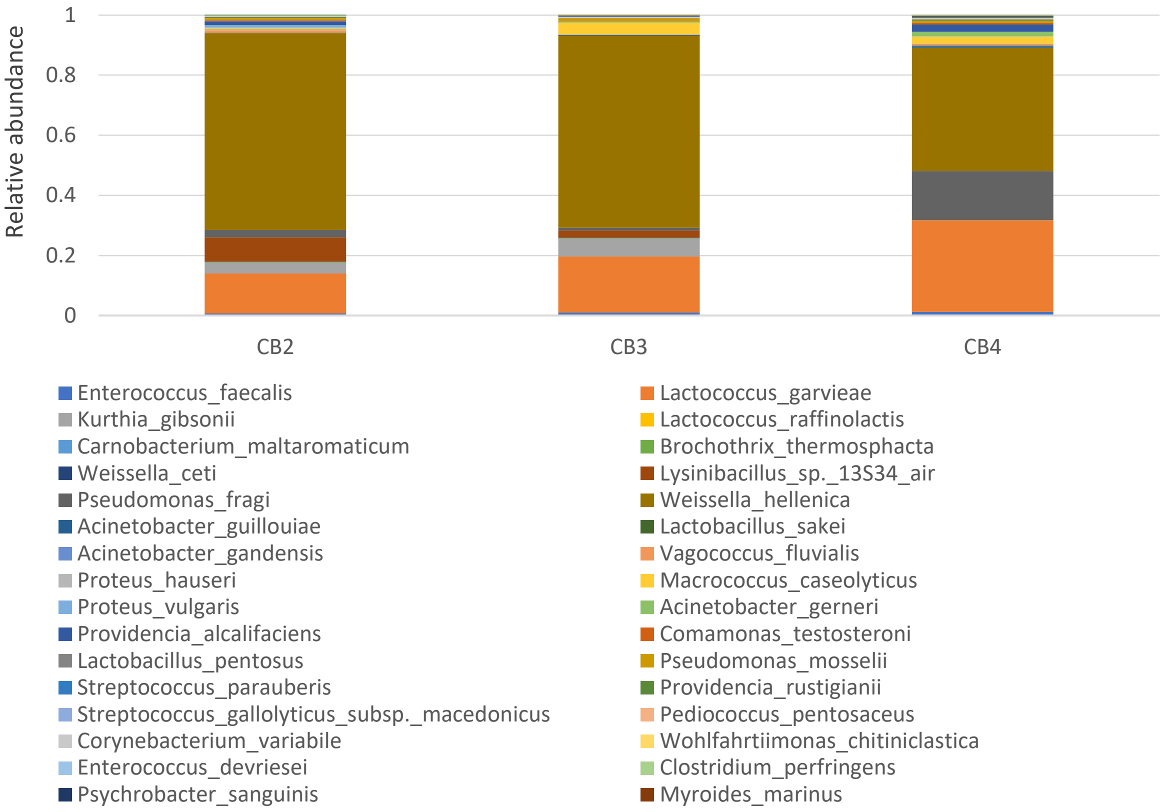

Figure S2: Taxonomic community profiles (at a genus level) of the swab samples following the second (CB2), third (CB3), and fourth (CB4) cleaning routine on the cutting board.

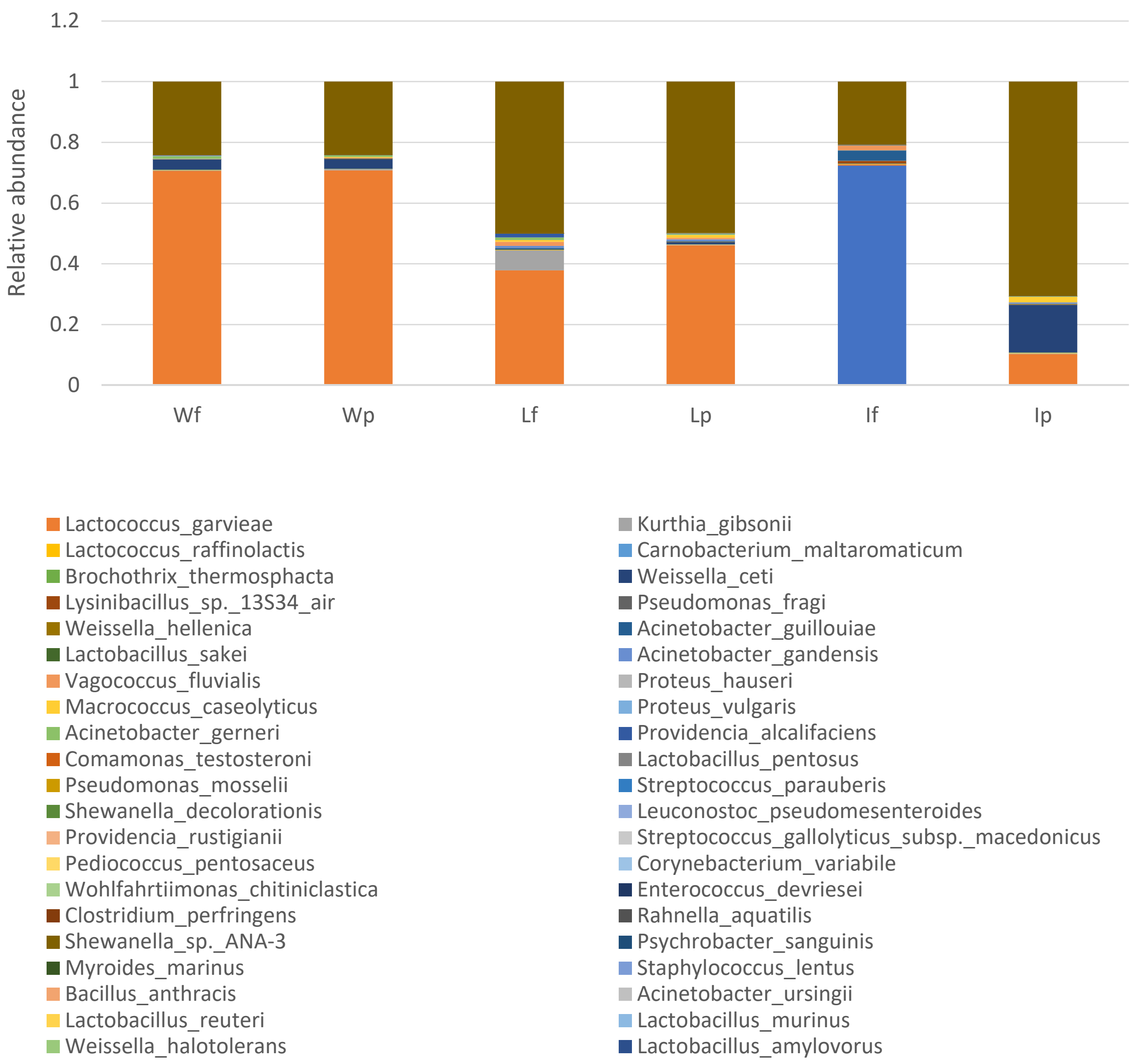

Figure S3: Relative abundance profiles of meat samples (at a genus level) with Lf, If, and Wf representing the unprocessed local meat, unprocessed imported meat, and unprocessed wet market meat, respectively. LP, Ip, and Wp represents the processed local meat, processed imported meat, and processed wet market meat, respectively.
